# Incidence and risk factors for anastomotic bleeding in lower gastrointestinal surgery

**DOI:** 10.1186/s13104-019-4403-0

**Published:** 2019-07-03

**Authors:** Jocelyne Hébert, Sherif Eltonsy, Jeffrey Gaudet, Caroline Jose

**Affiliations:** 1 Vitalité Health Network, Moncton, Canada; 2 Centre de Formation médicale du Nouveau-Brunswick, Moncton, Canada; 3 Maritime SPOR SUPPORT Unit, Moncton, Canada; 4 College of Pharmacy, Rady Faculty of Health Sciences, University of Manitoba, 750 McDermot Avenue, Winnipeg, MB R3E 0T5 Canada; 5 Université de Sherbrooke, Quebec City, Canada

**Keywords:** Anastomotic bleeding, Gastrointestinal Surgery, Postoperative, Complications, Hemorrhage

## Abstract

**Objective:**

Although major anastomotic bleeding after lower gastrointestinal surgery is considered rare, it can be life-threatening if not properly managed. The objective of this study was to assess the incidence of postoperative lower gastrointestinal intraluminal bleeding and to identify its potential risk factors. This retrospective cohort study used data from charts of 314 patients who underwent digestive surgery of the colon or small intestine. Details are reported for their sociodemographic data, surgical approach, comorbidities, timing and presentation of intraluminal bleeding events, bleeding diagnosis, treatment strategies, hospital length of stay, and clinical complications.

**Results:**

A total of 7 patients (2.3%) experienced intraluminal bleeding in the postoperative period. The average length of hospital stay before discharge was 12 days (median = 13 days). Patients with intraluminal bleeding had a significantly higher percentage of coronary artery diseases compared to patients without intraluminal bleeding (P value = .04), as well as having a cancer diagnosis (P value = .02). The clinical complications that were more likely in patients with intraluminal bleeding included requiring blood transfusions (P value = .01), reduction in hemoglobin (P value = .001), cardiac ischemia (P value = .02), and atrial fibrillations (P value = .02).

**Electronic supplementary material:**

The online version of this article (10.1186/s13104-019-4403-0) contains supplementary material, which is available to authorized users.

## Introduction

Among the most common lower gastrointestinal anastomosis complications are leakage and dehiscence, strictures, fistulas and bleeding [1, 2]. While risk factors for leakage and dehiscence, strictures and fistulas are widely reported [3–6], risk factors for anastomotic bleeding are not as well known. Most cases of postoperative anastomotic bleeding are self-limiting and are not commonly reported by surgeons, however major anastomotic bleeding can be life-threatening if not treated promptly [7]. The reported incidence rates for severe lower gastrointestinal bleeding range from .5 to 4.2% [8–11]. Severe lower gastrointestinal bleeding can be difficult to locate, making the diagnostic and therapeutic maneuvers challenging [11]. Conservative approaches have been successful in some cases, though only a handful have been reported in the literature [8]. Surgical techniques have evolved quickly, with newer generations of innovative medical and surgical materials being currently employed. Those advances render studies on lower gastrointestinal bleeding prevalence and management strategies relatively outdated, and novel evidence from recent studies become crucial and necessary.

Given the scarce evidence in the published literature on the risk factors associated with major anastomotic bleeding, this study aimed at assessing the incidence of postoperative lower gastrointestinal intraluminal bleeding and identifying its potential risk factors.

## Main text

### Methods

We conducted a single-center retrospective cohort study. The cohort was constructed using data from the reviewed charts of 314 consecutive patients who underwent digestive surgery of the colon or small intestine by six different surgeons. The procedures were performed between December 2009 and December 2014 at the Dr. Georges-L.-Dumont University Hospital Centre, Moncton, New Brunswick, Canada. The study cohort included low rectal anterior resection in 153 patients, right standard/extended hemicolectomy in 102 patients, small bowel resection in 22 patients, Ileocecal resection in 19 patients, partial colectomy in 6 patients, total colectomy and ileorectal anastomosis in 6 patients, and left hemicolectomy in 6 patients. Fifty-six percent of surgeries were performed by laparoscopy or assisted by laparoscopy compared to forty-four percent being open surgeries. The vast majority of anastomosis were fashioned using staples. An end to end type anastomosis was the technic used during all low anterior resections and some partial colectomies while a side to side anastomosis was used for most right hemicolectomies and small bowel resections as well as for a few partial colectomies. An end to side ileorectal anastomosis was most often used for cases of total colectomies.

Patients who experienced postoperative intraluminal bleeding were identified, and a comprehensive chart review of their clinical and operative notes was undertaken. Postoperative intraluminal bleeding was defined as a persistent rectorrhagia or melena occurring within 30 postoperative days and associated with a 20 g/L decrease in hemoglobin, and cases were confirmed after thorough medical chart review (Additional file 1: Fig. S1). Details were recorded for the following parameters: sociodemographic data, surgical approach, comorbidities, timing and presentation of intraluminal bleeding event, bleeding diagnosis, treatment strategies, hospital length of stay and clinical complications.

Descriptive statistics for the characteristics of patients were calculated and compared between patients with and without intraluminal bleeding. To account for the small sample size of patients with intraluminal bleeding, non-parametric tests, Fisher’s exact or Mann–Whitney U univariate analysis tests, were used to examine the difference for categorical and continuous variables, respectively. We reported the data as means ± standard deviations (SD) and percentages, as well as the calculated P value. A level of P < .05 was considered as statistically significant. Statistical analyses were conducted using SAS software, version 9.4 (SAS Institute Inc., Cary, NC). This study was approved by the Research Ethics Committee of the Vitalité Health Network. Consent to participate was waived, as only retrospective and de-identified data were used.

### Results

The operative morbidity data were reliably available for 309 patients who had undergone digestive surgery of the colon or small intestine and who were finally included in the cohort analysis. Forty-nine percent of patients were women, with an average age of 64 years (SD = 14.2) and average body mass index (BMI) of 28.6 (SD = 6.8). The most commonly used preoperative prophylaxis medications consisted of Heparin prophylaxis (64.1%). Some patients were on antiplatelets (26.2%), and natural products (14.2%) preoperatively. Whereas the most common postoperative medications were Heparin prophylaxis (87.7%), Lovenox (9.4%), antiplatelets (19.1%), and Nonsteroidal Anti-Inflammatory drugs (NSAIDs) (22.9%).

A total of 7 (2.3%) patients experienced intraluminal bleeding in the postoperative period (Table 1). Overall, patients with and without intraluminal bleeding were comparable in their sociodemographic and clinical variables measured at admission. Tests of differences between groups confirmed the similarity between groups (i.e. P values ≥ .05), with the exception of two variables. Among the recorded comorbidities, patients with intraluminal bleeding had a significantly higher percentage of coronary artery diseases compared to patients without intraluminal bleeding (P value = .04). Moreover, having a cancer diagnosis as a motive for surgery was significantly higher among patients with intraluminal bleeding (P value = .02). No other differences were found between groups on the following operation related variables: length of hospital stay, difficulties in per-operative, surgical approach and type of mounting.

**Table 1:** Characteristics and univariate analysis of patients with and without intraluminal bleeding included in the analysis

Demographics	With intraluminal bleeding (n = 7)	Without intraluminal bleeding (n = 302)	Fisher’s exact or Mann–Whitney U test
	No. (%) or Mean ± SD	No. (%) or Mean ± SD	P value
Sex (female)	2 (28.6)	148 (49.0)	.45
Age (years)	69 ± 7.4	64 ± 14.3	.33
Body Mass Index (BMI)	27.2 ± 3.6	28.6 ± 6.9	.58
Comorbidity			
History of cancer	1 (14.3)	45 (14.9)	.99
Coronary artery disease	*3 (42.9)*	*34 (11.3)*	*.04*
Peripheral vascular disease	0 (0)	24 (7.8)	.56
Hypertension	3 (42.9)	139 (46)	.99
Previous surgery	2 (28.6)	127 (42.1)	.70
Surgery motive			
Cancer	*7 (100)*	*169 (55.9)*	*.02*
Diverticulosis	0 (0)	55 (18.2)	.36
Other	0 (0)	78 (25.8)	.20
Surgical approach			
Laparoscopic	2 (28.6)	144 (47.6)	.45
Laparotomy	3 (42.9)	133 (44.0)	.99
Laparoscopy assisted surgery	2 (28.6)	25 (8.3)	.12

Italic values indicate significance of the p value (p < 0.05)

The characteristics and clinical courses of patients with intraluminal bleeding are presented in Table 2. The average time from primary surgery to intraluminal bleeding was 6 days and ranged from 1 to 10 days, however most of the patients (i.e. 5 patients [71.4%]) had their postoperative bleeding 6 days or more after surgery. Bleeding symptoms included rectorrhagia in 5 patients and melena in the other 2 cases. Four patients needed packed red blood cell transfusions (2, 3, 4 and 6 units) and one patient needed operative treatment. In the six cases of non-operative treatment, clinical and endoscopy investigations were performed to confirm the origin of the bleeding site. Surgery was required for the other patient due to the persistent bleeding and haemodynamic instability. The average length of hospital stay before discharge was 12 days (median = 13 days), ranging between 4 and 26 days.

**Table 2:** Characteristics and clinical courses of patients with intraluminal bleeding

Patient	Age	Gender	Diagnosis	Primary surgical technique	Surgical approach	Anastomosis technique*	Time to postoperative hemorrhage (days)	Bleeding diagnosis	Treatment	Hospital stay (days)
1	73	M	Non-occlusive cancer	Total colectomy and ileorectal anastomosis	Laparotomy	Side to side, mechanical	PO #6	Clinical		26
2	81	F	Non-occlusive cancer	Low rectal anterior resection	Laparoscopy assisted	End to end, mechanical	PO #7	Endoscopic	Transfusions (2);	13
3	66	M	Non-occlusive cancer	Right standard/extended hemicolectomy	Laparoscopic	Side to side, mechanical	PO #1	Clinical	Transfusions (3);	14
4	72	M	Non-occlusive cancer	Right standard/extended hemicolectomy	Laparoscopy assisted	Side to side, mechanical	PO #10	Endoscopic	Transfusions (4);	13
5	57	M	Cancer (Metastasis melanoma)	Small bowel resection	Laparotomy	Side to side, mechanical	PO #10	Radiological	Transfusions (6); re-operation	8
6	66	M	Cancer (unresectable polyp)	Right standard/extended hemicolectomy	Laparoscopic	Side to side, mechanical	PO #3	Clinical		4
7	70	F	Non-occlusive cancer	Low rectal anterior resection	Laparotomy	End to end, mechanical	PO #7	Clinical		7

*PO* postoperative

* Side to side anastomosis technique: one application of linear cutter stapler on the anti-mesenteric side of both bowel ends and one application of a linear stapler to close the end. End to end anastomosis technique: one application of a circular stapler

Several clinical complications were more likely in patients with intraluminal bleeding (Table 3). These complications included a greater likelihood of requiring blood transfusion (P value = .01), experiencing a drop in hemoglobin greater than 20 g/dL (P value = .001), experiencing cardiac ischemia (P value = .02), and suffering an atrial fibrillation (P value = .02).

**Table 3:** Clinical complications among patients with and without intraluminal bleeding

	With intraluminal bleeding (n = 7)	Without intraluminal bleeding (n = 302)	Fisher’s exact or Mann–Whitney U test
	No. (%)	No. (%)	P value
Transfusion	*4 (57.1)*	*37 (12.3)*	*.01*
Decrease in hemoglobin ≥ 20 g/dL	*6 (85.7)*	*32 (10.6)*	*.001*
Re-intervention	1 (14.3)	15 (4.9)	.31
Wound infection	2 (28.6)	27 (8.9)	.13
Cardiac ischemia	*2 (28.6)*	*8 (2.7)*	*.02*
Pulmonary overload	1 (14.3)	2 (.66)	.07
Atrial fibrillation	*2 (28.6)*	*8 (2.7)*	*.02*
Septic shock	1 (14.3)	6 (2.0)	.15
Gastroparesis	1 (14.3)	2 (.66)	.07
Acute renal failure	1 (14.3)	10 (3.3)	.23
Death	0 (0)	9 (2.9)	.23

Italic values indicate significance of the p value (p < 0.05)

### Discussion

In this study of patients who underwent digestive surgery of the colon or small intestine, we observed a moderate incidence of intraluminal bleeding in the postoperative period (2.3%) compared to previously published reports [7–9, 12]. Most of the bleeding cases occurred within 6 days postoperatively and patients were hospitalized for 12 days on average before discharge. From the comparison tests conducted, we observed a significantly increased proportion of coronary artery diseases and cancer diagnosis among patients with intraluminal bleeding compared to patients without intraluminal bleeding. Moreover, significant increases in some clinical complications were observed, including blood transfusions, decrease in hemoglobin ≥ 20 g/dL, cardiac ischemia and atrial fibrillations.

The published evidence on lower gastrointestinal anastomosis and postoperative bleeding is limited [7–9, 12]. Defining anastomotic hemorrhage as a massive bleeding occurrence that needed endoscopic or surgical intervention, Tanizawa et al. reported an incidence of .4% and a mean of 24 days before discharge [7]. In the study, none of the baseline variables (i.e. age, sex, use of anticoagulants and hemoglobin level) was significantly different between the groups with and without anastomotic hemorrhage [7]. In a study on the risk factors for delayed bleeding after colorectal endoscopic submucosal resection, delayed bleeding occurred in 6.6% of the patients and there was no significant difference between the two groups in mean age, sex and current use of antithrombotic agents [12]. Moreover, the study demonstrated that the lesion location in the rectum was a significant risk factor for delayed bleeding [12]. Severe lower gastrointestinal bleeding was reported also in two case-series studies [8, 9]. The reported incidence of bleeding and median length of hospital stay were .5% and 11 days among 1389 patients [8] and .8% and 7 days among 777 patients [9], respectively.

Respecting specific technical details while creating the anastomosis are known to reduce the incidence of anastomotic bleeding [13, 14]. A latero-lateral anastomosis should be fashioned by using the anti-mesenteric borders of the bowel and the stapled GIA line should be inspected for bleeding [14]. On an end-to-end anastomosis, clearing the mesentery from the transected bowel edge reduces the risk of having a major vessel left untied and stapled with the anastomosis. All anastomotic bleeding sites should be ligated with a absorbable stitch and not cauterized [14]. Even when these technical details are respected, major anastomotic bleeding will occur in .5–4.2% of lower gastrointestinal anastomosis [8, 9]. Our study had a 3.4% anastomotic bleeding rate in proximal side to side anastomosis versus a 1.3% rate in end to end anastomosis. Proximal side to side anastomosis are slightly more challenging to approach by endoscopy. There is less visibility with a blood covered bowel and the longer insulation time increases the risk of perforation. Our study could not demonstrate this increase in complication rate with endoscopic treatment, mainly due to the small sample size and the absence of a necessary endoscopic intervention.

The primary objective of the current study included the identification of potential risk factors associated with major anastomotic bleeding. We found significantly increased proportions of coronary artery diseases and cancer diagnosis among patients with intraluminal bleeding. Additional analyses using regression models were performed but not reported due to small sample sizes and convergence issues. Future studies are warranted to carefully investigate those two potential risk factors in larger patient populations. Moreover, additional investigations into the observed major complications (i.e. cardiac ischemia and atrial fibrillations) are required to help guide physicians through optimal approaches in managing patients after surgery.

## Limitations

Results of the current study has to be viewed cautiously given the presence of some important limitations that impact the study conclusions. Due to the retrospective and nonrandomized nature of the study, we were unable to reduce bias that could be caused by unmeasured factors. The sample size of the study represents another major limitation. We did not have enough power to report the regression analyses models due to the relative rarity of the outcome of interest (only 7 cases), rendering our models unstable and affecting their validity. We were only able to evaluate the clinical events if they were documented in the chart by the physician; potentially underreporting some clinical events. Although unlikely because of the small community served, we were unable to confirm that no other patient presented with intraluminal bleeding at another hospital since our data predates the provincial electronic health records. Given the mentioned limitations, additional studies are essential to examine if the results of the current study can be corroborated in other cohorts of larger size. Finally, the data were retrieved from one center, affecting the generalizability of our results.

## Additional file

**Additional file 1: File S1.** Cohort selection flowchart.

## Data Availability

The datasets used and/or analysed during the current study are available from the corresponding author on reasonable request.

## Abbreviations

SD: standard deviations
BMI: body mass index
NSAIDs: Nonsteroidal Anti-Inflammatory drugs
