# Supplementary material for: Incidence and risk factors for anastomotic bleeding in lower gastrointestinal surgery
Source: BMC Res Notes. 2019 Jul 3;12:378. doi: 10.1186/s13104-019-4403-0 (PMC6607592; doi:10.1186/s13104-019-4403-0)
Supplement: Supplementary file 1 — Additional file 1: File S1. Cohort selection flowchart. [file 13104_2019_4403_MOESM1_ESM.docx]

**302** patients without intraluminal bleeding

**7** cases of intraluminal bleeding

- Identified as rectorrhagia or melena and 20g/L decrease in hemoglobin
- Confirmed by medical chart review

**5** excluded patients with pre-existing bleeding or required multiple anastomosis

**309** patients included in the analysis

**314 eligible patients at study inception who underwent digestive surgery of the colon or small intestine**

**Figure S1.** Cohort selection flowchart
